# Bridging the theory-practice gap in laboratory medicine: development and application of a standardized case library for fostering clinical reasoning in postgraduate training

**DOI:** 10.3389/fmed.2026.1834799

**Published:** 2026-06-24

**Authors:** Qin Wang, Jingjing Zhang, Hao Zhang, Aihua Wang, Yuanyuan Wu, Zhi Duan, Zhidong Chen, Tingdong Zhou, Yu Wei, Wenwen Chu, Qiang Zhou

**Affiliations:** 1 Department of Clinical Laboratory, The Second Affiliated Hospital of Anhui Medical University, Hefei, China; 2 Department of Geriatric Cardiology, The Second Affiliated Hospital of Anhui Medical University, Hefei, China

**Keywords:** case-based learning, clinical laboratory diagnostics, clinical thinking, postgraduate education, teaching case library

## Abstract

**Background:**

Case-based learning (CBL) is crucial for developing clinical reasoning in medical education. However, its application in clinical laboratory diagnostics is hindered by the absence of standardized, real-world teaching cases. This study aimed to develop a comprehensive teaching case library and conduct an exploratory evaluation of its potential associations with student outcomes.

**Methods:**

This study employed a two-phase, quasi-experimental, non-randomized design with a historical control. In the first phase, a standardized case library was constructed using real patient data from a tertiary hospital’s Laboratory and Hospital Information Systems, following principles of systematic coverage and clinical relevance. All 28 cases, spanning six laboratory subspecialties, underwent dual review by laboratory medicine specialists and clinicians. In the second phase, this library was integrated into a structured, closed-loop CBL curriculum. A total of 36 professional master’s degree students in Clinical Laboratory Diagnostics were included. The historical control group (*n* = 18, enrolled 2019–2022) received traditional lecture-based teaching, while the CBL group (*n* = 18, enrolled 2023–2025) received CBL instruction. The two groups had comparable baseline characteristics, including age, gender distribution, and previous academic performance (all *P* > 0.05). Teaching effectiveness was assessed through a mixed-methods approach, including comparative analysis of examination scores, classroom participation rates, and thematic analysis of qualitative student feedback.

**Results:**

The library comprises 28 peer-reviewed cases. Compared to the historical control group, the CBL group showed higher engagement levels: instructor ratings showed that 66.6% of CBL students achieved “high engagement” (score ≥ 4 on a 5-point scale) versus 33.3% in the control group; student self-reported mean composite engagement score was 4.5 ± 0.4 in the CBL group compared to 3.2 ± 0.6 in the control group (*P* < 0.01), representing a 1.3-point increase. Additionally, the CBL group showed a mean score improvement of 5.8 points on end-of-rotation examinations (95.4 ± 2.9 vs. 89.6 ± 7.5, *P* = 0.004). Thematic analysis of student feedback revealed that CBL was perceived to reduce cognitive load during the theory-practice transition, enhance professional self-confidence, and foster a stronger sense of professional identity.

**Conclusion:**

A systematically constructed and closed-loop implemented case library, integrated with CBL, appears to offer a promising approach for bridging the theory-practice gap and supporting the development of essential clinical competencies. These preliminary, hypothesis-generating findings from a single-center, quasi-experimental study should be interpreted as exploratory rather than confirmatory, and they warrant validation in larger-scale, preferably randomized, investigations. This scalable educational model offers a practical framework for laboratory medicine training and holds potential for improving interprofessional collaboration.

## 1 Introduction

Clinical laboratory diagnostics is a secondary discipline under clinical medicine. Its core responsibility is to provide accurate and reliable laboratory data, offering scientific evidence and essential support for critical clinical decisions, including disease diagnosis, therapeutic effect evaluation, prognosis assessment, and health management. As such, one of the primary objectives of professional master’s degree education in clinical laboratory diagnostics is to cultivate laboratory physicians with integrated competencies in clinical laboratory practice and scientific research innovation (1, 2). The essence of clinical practice teaching lies in systematically developing students’ clinical laboratory thinking, with particular emphasis on enhancing their ability to accurately interpret laboratory results and solve complex practical problems in clinical laboratory settings (3).

In the context of clinical laboratory diagnostics, clinical reasoning takes on a distinctive form. Unlike direct patient-facing specialties, laboratory physicians engage in a unique mode of reasoning that involves: (1) interpreting numerical data in light of pre-analytical, analytical, and post-analytical variables; (2) integrating disparate laboratory findings to generate diagnostic hypotheses that guide clinicians; (3) recognizing patterns across multiple test results that may indicate specific pathologies; and (4) evaluating the clinical utility of emerging biomarkers while avoiding over-diagnosis or misinterpretation. Thus, clinical reasoning in laboratory medicine is not merely about reading test results but about formulating an integrated diagnostic interpretation from complex, multi-dimensional laboratory data. Its importance in this particular context cannot be overstated: erroneous or incomplete laboratory reasoning can lead to missed diagnoses, unnecessary testing, inappropriate treatment, and ultimately patient harm. Therefore, systematically cultivating clinical reasoning skills is a core educational mandate for laboratory medicine training programs.

Case-based learning (CBL) has emerged as a highly regarded and widely promoted teaching method in medical education in recent years. This model adheres to the core principles of “being grounded in real clinical cases, guided by scientific questions, student-centered, and teacher-facilitated,” demonstrating advantages in stimulating learning initiative and strengthening the integration of theory and practice (4, 5). Implementing case-based teaching is an important approach to reinforcing the cultivation of practical abilities and the development of clinical thinking among professional degree postgraduates (6). By engaging with authentic cases, students not only acquire knowledge but also learn to navigate uncertainty, weigh evidence, and communicate effectively—skills that are essential for competent clinical practice. Thus, case-based learning is particularly well-suited to fostering clinical reasoning in laboratory medicine because it places students in realistic scenarios where they must actively apply cognitive processes—such as hypothesis generation, data interpretation, and evidence synthesis—rather than passively memorizing facts.

However, in the teaching practice of clinical laboratory diagnostics, the application of the CBL model still faces practical challenges, such as the lack of standardized teaching cases and inconsistent teaching implementation processes and evaluation criteria (7). These factors constrain further improvements in teaching quality. To address the gap in standardized teaching materials and inconsistent CBL implementation, this study aimed to systematically construct a comprehensive teaching case library for Clinical Laboratory Diagnostics and to conduct an exploratory evaluation of its potential association with student outcomes among professional master’s degree students through a structured CBL curriculum, with the understanding that findings would be hypothesis-generating rather than confirmatory.

## 2 Materials and methods

### 2.1 Study design and setting

This study was conducted in two phases: first, the development of a teaching case library for Clinical Laboratory Diagnostics, followed by its implementation within a case-based learning (CBL) curriculum for professional master’s degree students. The research was carried out at the Department of Clinical Laboratory, The Second Affiliated Hospital of Anhui Medical University.

### 2.2 Phase 1: development of the teaching case library

#### 2.2.1 Case source and selection criteria

Potential teaching cases were initially identified through a retrospective search of the Hospital Information System (HIS) and Laboratory Information System (LIS). The selection process prioritized real, complete clinical cases that were characteristic of common or complex diseases and contained rich, multifaceted laboratory data. Each candidate case underwent a dual review process by a senior laboratory medicine specialist and a clinical physician to confirm its educational suitability. Final inclusion in the library was contingent upon a confirmed diagnosis, comprehensive clinical documentation (including chief complaint, medical history, and physical examination findings), and a complete set of relevant laboratory results.

#### 2.2.2 Data anonymization and privacy protection

To ensure patient privacy, all clinical data retrieved from HIS/LIS underwent strict anonymization prior to case development. The following measures were implemented: (1) removal of all direct identifiers (name, medical record number, ID number, phone number, address, exact date of birth); (2) de-identification of indirect identifiers (admission/discharge dates generalized to month/year, physician names removed); (3) assignment of unique non-identifiable case codes (e.g., IMM-01); (4) data aggregation for rare diseases (e.g., reporting age as ranges); (5) manual redaction of free-text comments; and (6) no direct patient contact. A random sample of 20% of cases was independently reviewed to verify that no residual patient identifiers remained. These procedures complied with Chinese privacy laws, HIPAA de-identification standards, and the Declaration of Helsinki.

#### 2.2.3 Case development team

The cases were developed by multidisciplinary team, the composition of which is shown in Table 1.

**TABLE 1:** Composition of the multidisciplinary team.

Role	Number	Qualifications	Responsibilities
Senior laboratory medicine specialists	3	≥ 10 years of clinical and teaching experience	Case selection, laboratory data interpretation, content review
Clinical physicians	2	≥ 10 years of clinical experience	Clinical context validation, diagnostic accuracy review
Medical education experts	2	≥ 8 years of experience in CBL curriculum	Pedagogical design, learning objective alignment, question formulation
Graduate teaching assistants	3	≥ 3 years of clinical experience (Laboratory Physician)	Case formatting, literature retrieval, reference compilation

And the lead author coordinated the overall development process. Each case was assigned to a primary developer from the laboratory medicine team, who drafted the initial case content using real patient data, then iteratively refined it based on feedback from the clinical physicians and education experts.

#### 2.2.4 Case standardization

A standardized case template was developed to unify the structure of all cases. The template included the following fixed sections: (1) Metadata (ID, title, subspecialty, type, difficulty, duration); (2) Learning Objectives; (3) Clinical Case Data (complaint, history, PE, laboratory data, other ancillary tests); (4) Guided Discussion Questions; (5) Teaching Implementation Flow; (6) Key Takeaways and References; (7) Validation and Management Information; (8) Appendices (code, difficulty index, usage instructions) (Supplementary Material S1)

#### 2.2.5 Case validation

The validation process consisted of three levels. First, content validity was assessed by a panel of five experts (three senior laboratory medicine specialists and two clinicians), who reviewed each case against predefined criteria: clinical authenticity, educational relevance, alignment with learning objectives, and clarity of presentation. Cases were rated on a 4-point Likert scale (1 = unacceptable, 4 = excellent), and only cases with a mean rating ≥ 3.5 were retained. Second, pedagogical validity was evaluated by three medical education experts, who assessed whether the case design appropriately promoted clinical reasoning and aligned with CBL principles. Third, face validity was confirmed through pilot testing with a small group of graduate students (*n* = 3–5), who reviewed the cases for clarity, feasibility, and engagement. Feedback from pilot testing was incorporated to refine case materials before final inclusion.

#### 2.2.6 Difficulty index control

To ensure that cases were appropriately challenging for the target learner level (professional master’s degree students), a preliminary difficulty index was estimated for each case using a modified version of the case difficulty formula adapted from medical education literature. The index incorporated four parameters: (1) clinical complexity (number of differential diagnoses); (2) data volume (number of laboratory parameters to interpret); (3) cognitive demand (level of integration required); and (4) prior knowledge required (based on curriculum mapping). Each parameter was rated on a 3-point scale (1 = low, 3 = high). The difficulty index for each case was calculated as the mean score of the four parameters, yielding a possible range of 1.0–3.0 (Table 2). And cases were categorized into three difficulty tiers: easy (mean score 1.0–1.5), moderate (1.6–2.2), and difficult (2.3–3.0).

**TABLE 2:** Parameters and scoring criteria for difficulty index.

Parameter	Scoring Criteria	1 (Low)	2 (Moderate)	3 (High)
Clinical complexity	Number of differential diagnoses	1–2	3–4	≥ 5
Data volume	Number of laboratory parameters to interpret	≤ 5	6–10	≥ 11
Cognitive demand	Level of integration required	Simple description	Comprehensive analysis	Critical evaluation
Prior knowledge required	Based on curriculum mapping	Foundational knowledge	Intermediate knowledge	Advanced/cutting-edge knowledge

Difficulty Index = (Sum of four parameter scores) ÷ 4.

#### 2.2.7 Case categorization and distribution

The selected cases were systematically organized into six core modules mirroring the main subspecialties of laboratory medicine: Clinical Hematology (6 cases), Clinical Body Fluids Analysis (5 cases), Clinical Biochemistry (6 cases), Clinical Immunology (5 cases), Clinical Microbiology (3 cases), and Clinical Molecular Biology (3 cases). All 28 cases were utilized during the CBL curriculum, with each case covered in a dedicated 90-min session (in actual teaching, some cases may be combined for discussion). The distribution was designed to reflect the relative instructional emphasis of each subspecialty in the overall curriculum.

### 2.3 Phase 2: implementation of case-based learning (CBL)

#### 2.3.1 Study design

A quasi-experimental, non-randomized design with a historical control group was employed in this study. Due to the natural progression of curriculum reform and the infeasibility of random assignment within a single cohort, a historical control group was employed. This design allowed for the evaluation of the new CBL curriculum while maintaining the integrity of the existing educational program. The historical control group was drawn from the preceding cohort, during which the traditional teaching method was used, with all other curricular elements (e.g., course content, assessment format, faculty) kept consistent.

##### 2.3.1.1 Allocation

Participants were not randomized to groups due to operational constraints of the educational setting. Instead, group assignment was determined by enrollment cohort, which served as a natural partition.

##### 2.3.1.2 Temporal direction

This was a retrospective comparison using prospectively collected data for the experimental group. Outcome data for the historical control group were extracted from existing academic records, while outcome data for the experimental group were collected prospectively during the 2023–2025 academic years.

##### 2.3.1.3 Rationale for quasi-experimental design

Random assignment of students to different teaching methods was not feasible because: (a) using different teaching methods simultaneously would create cross-contamination between groups and (b) withholding a potentially superior educational intervention from a randomly assigned subgroup would raise ethical concerns. The historical control design was therefore the most practical and ethically acceptable approach.

##### 2.3.1.4 Internal validity considerations

To minimize selection bias and enhance comparability between groups, the following measures were implemented:

- Both groups were drawn from the same training program at the same institution.
- Admission criteria and curriculum content (except for the teaching method) were identical.
- Baseline comparability was confirmed for age, gender distribution, and prior academic performance (all *P* > 0.05).
- End-of-rotation examinations of similar difficulty, taken from the same item bank, were used for both groups.
- Grading was performed by faculty members blinded to the study hypothesis.

#### 2.3.2 Sample size

Given the quasi-experimental, non-randomized design with a historical control group, a conventional a priori sample size calculation based on randomization was not applicable. The sample size was census-based, including all eligible professional master’s degree students enrolled in the Clinical Laboratory Diagnostics program at our institution during the study period (2019–2025), resulting in a total of 36 students (*n* = 18 per group). This sample size is comparable to many published educational intervention studies in specialized postgraduate training programs.

#### 2.3.3 Participants

Participants were eligible for inclusion in this study if they met the following criteria:

- Enrolled as a professional master’s degree student in the Clinical Laboratory Diagnostics program at our institution
- Enrolled during the study period (September 2019 to September 2025)
- Completed the full rotation in the Department of Clinical Laboratory
- Completed all required end-of-rotation examinations
- Provided written informed consent (experimental group) or had existing academic records available for retrospective analysis (historical control group)

Participants were excluded from this study if they met any of the following criteria:

- Incomplete training due to leave of absence, transfer, or withdrawal from the program
- Missing end-of-rotation examination scores
- Prior exposure to CBL methodology
- Declined to provide informed consent

A total of 36 professional master’s degree students majoring in Clinical Laboratory Diagnostics were included, with 18 in the historical control group (enrolled 2019–2022) who received traditional lecture-based teaching and 18 in the experimental group (enrolled 2023–2025) who received CBL. Baseline characteristics including age at enrollment, gender, and prior academic performance were collected from archived admission records.

#### 2.3.4 Facilitator standardization and training

Given that case-based learning (CBL) requires active facilitation to guide student discussion and clinical reasoning, all facilitators involved in the experimental group underwent a structured standardization and validation process to ensure consistency in implementation.

##### 2.3.4.1 Facilitator selection

All facilitators were senior laboratory medicine specialists with: (1) ≥ 5 years of clinical teaching experience; (2) Prior familiarity with CBL pedagogy (attended at least one CBL workshop prior to the study).

##### 2.3.4.2 Standardized training protocol

Prior to the start of the CBL curriculum, all facilitators completed a 6-h standardized training program consisting of the following modules (Table 3).

**TABLE 3:** Modules of the standardized facilitator training program.

Module	Duration	Content
Module 1: CBL principles	2 h	Philosophy of CBL; differences from traditional teaching; facilitator role vs. lecturer role
Module 2: case library familiarization	1.5 h	Review of all cases; learning objectives; guided questions; key teaching points
Module 3: facilitation techniques	1.5 h	Strategies for promoting student participation; managing group dynamics; Socratic questioning; providing feedback without giving answers
Module 4: role-play and simulation	1 h	Practice facilitation with mock CBL sessions using pilot cases; peer and expert feedback

##### 2.3.4.3 Facilitation standardization tools

To ensure consistent implementation across facilitators and sessions, the following standardization tools were provided (Table 4).

**TABLE 4:** Standardization tools to ensure consistency in teaching implementation.

Tool	Description	Purpose
Facilitator guide	A detailed manual for each case, including learning objectives, timing suggestions, key discussion points, common student misconceptions, and model answers to guided questions	Ensure all facilitators cover core content consistently
Structured session template	A standardized 90-min session plan (5-min introduction, 15 min per stage across 5 stages, 10-min summary)	Maintain consistent time allocation and flow
Facilitation checklist	A 10-item checklist of key facilitation behaviors (see Supplementary Material S2)	Monitor fidelity to CBL principles

#### 2.3.5 CBL teaching protocol

The CBL sessions were conducted using a structured three-stage process—preparation, in-class execution, and evaluation—which was applied uniformly across all cases.

##### 2.3.5.1 Pre-class preparation

One week prior to each session, students were provided with the relevant case materials and guiding questions. To support their self-directed learning, they were also assigned pertinent literature and current clinical guidelines.

##### 2.3.5.2 In-class implementation

**FIGURE 1:**
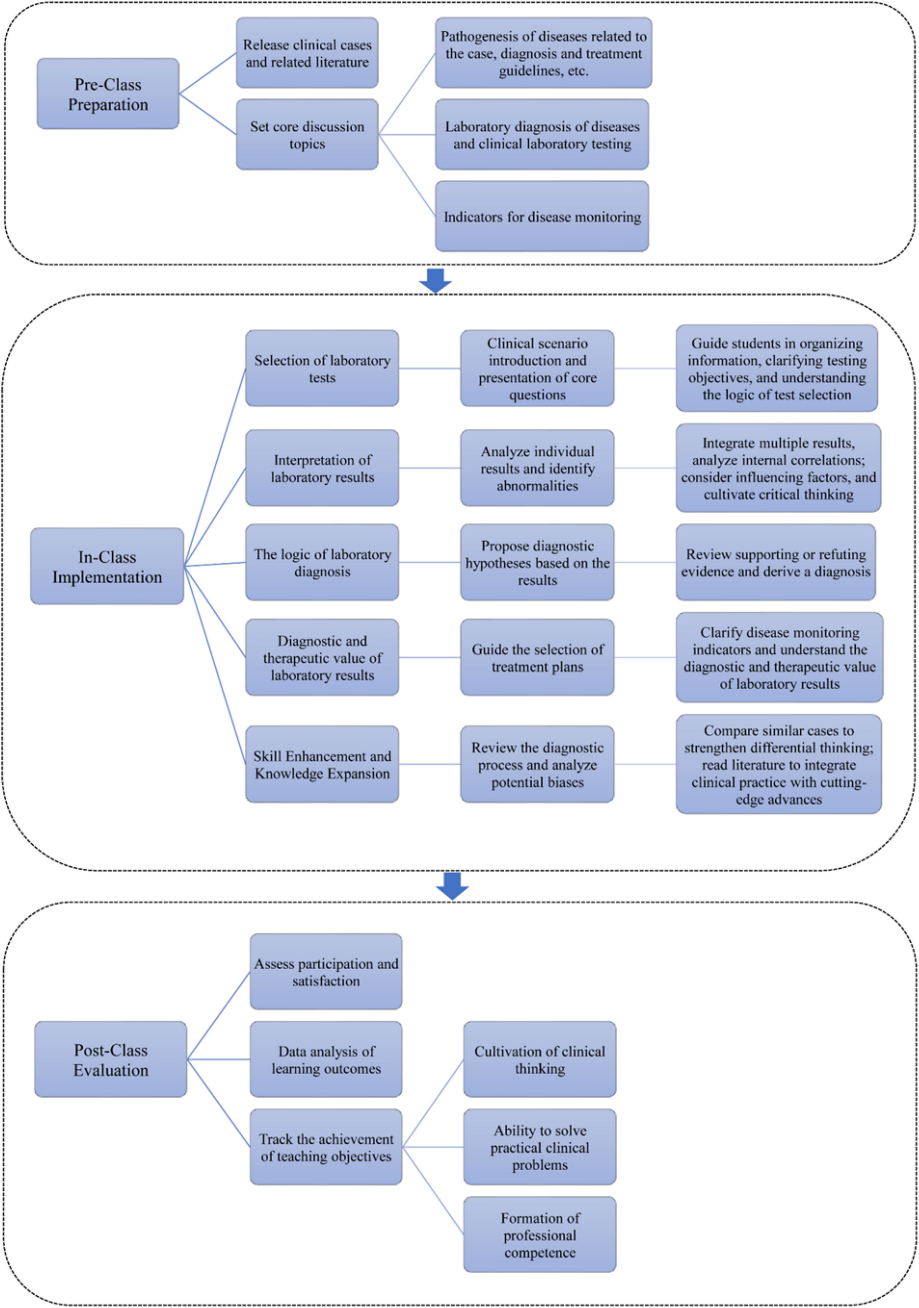
Implementation process of case-based teaching in clinical laboratory diagnostics.

Each in-class session was designed as a multi-stage, interactive process to guide students from foundational knowledge to advanced clinical reasoning. Students were divided into small discussion groups of 4–5 students per group to facilitate active participation and peer interaction. The session structure typically included: (1) correlating clinical presentations with appropriate laboratory investigations; (2) interpreting laboratory data to identify significant abnormalities; (3) formulating evidence-based diagnostic hypotheses; (4) discussing the utility of laboratory tests in therapeutic monitoring; and (5) exploring recent advances and relevant guidelines. A detailed illustration of this process using a representative case (systemic lupus erythematosus) is presented in the Results section (Figure 1).

##### 2.3.5.3 Post-class evaluation

Following each session, student learning outcomes, engagement, and satisfaction were assessed using structured questionnaires and a standardized teaching evaluation form.

#### 2.3.6 Traditional teaching protocol (historical control)

The historical control group received traditional lecture-based teaching covering the same core content. Traditional sessions were instructor-centered, with the teacher presenting didactic lectures on laboratory test interpretation, disease mechanisms, and diagnostic algorithms. Case discussions, if any, were limited to brief examples provided by the instructor rather than student-centered active learning. No structured case library or guided discussion questions were used.

### 2.4 Outcome measures

To evaluate the effectiveness of the implemented CBL approach, the following outcomes were assessed.

#### 2.4.1 Student engagement

Student engagement was evaluated using multi-method approach incorporating instructor ratings and student self-reports.

##### 2.4.1.1 Method 1: instructor rating of engagement

At the end of each session, the lead instructor rated the overall class engagement on a 5-point Likert scale (1 = very low, 2 = low, 3 = moderate, 4 = high, 5 = very high) based on the following global descriptors:

- High engagement (4–5): Most students actively participated; rich discussion with multiple perspectives
- Moderate engagement (3): Several students participated; discussion occurred but with limited diversity
- Low engagement (1–2): Few students participated; minimal discussion

##### 2.4.1.2 Method 2: student self-reported engagement

Following each CBL session, students completed a brief anonymous post-session survey designed to assess their self-perceived engagement. The survey comprised five quantitative items measured on a 5-point Likert scale (1 = strongly disagree, 5 = strongly agree), along with one open-ended question (Supplementary Material S3). A composite engagement score was calculated for each student as the mean of the five Likert-scale items (range 1–5), with higher scores indicating greater self-perceived engagement.

For the historical control group, a parallel version of the post-session survey (traditional lecture format) was used, with wording adapted to the lecture context (e.g., “lecture engagement,” “classroom interactions,” “lecture format,” “instructor”) (Supplementary Material S3).

#### 2.4.2 Academic performance

##### 2.4.2.1 Examination format and structure

The end-of-rotation examination was a comprehensive assessment administered to all students at the end of each training phase. The examination consisted of five sections with a total of 100 points (Table 5). The examination duration was 120 min.

**TABLE 5:** Composition and format of the end-of-rotation examination.

Section	Question type	Number of questions	Points per question	Total points	Description
Section A1	Single Best Answer Multiple Choice Questions (MCQs)	15	1	15	Single-sentence question assessing foundational concepts and factual knowledge
Section A2	Single best answer multiple choice question (MCQ) with clinical vignette	15	1	15	Brief clinical case presentation (2–4 sentences) requiring diagnostic reasoning and test interpretation
Section A3/A4	Case-based multiple-choice question (MCQ) series	15	1	15	Extended clinical case (1–2 paragraphs) with 2–4 related questions assessing sequential reasoning and integrated decision-making
Section B1	Extended matching question (EMQ)/Standard matching set	20	1.5	30	Several questions sharing a common set of 8–10 possible answers (diagnoses, tests, or interpretations); select the best match for each
Case analysis	Complex case analysis with multiple selection items	10	2.5	25	Complex clinical case (2–3 paragraphs) with multiple questions, requiring responses integrating clinical and laboratory data

##### 2.4.2.2 Validity of the examination

To ensure that the examination validly measured students’ clinical laboratory knowledge and reasoning skills, content validity was established by mapping the examination to the six core subspecialty modules in proportion to instructional hours. Four subspecialties—Clinical Hematology, Clinical Biochemistry, Clinical Immunology, and Clinical Microbiology—each received 20% of instructional hours and 20 examination points (20 points each). The remaining two subspecialties—Clinical Body Fluids and Clinical Molecular Biology—each received 10% of instructional hours and 10 examination points (10 points each). This ensured that the 100-point examination proportionally represented the curriculum’s instructional emphasis across all subspecialties.

A panel of five content experts (three senior laboratory medicine specialists and two clinicians, none of whom were involved in teaching the CBL group) reviewed all examination items for: (a) clinical relevance and accuracy, (b) alignment with learning objectives, (c) clarity and unambiguity of wording, and (d) appropriateness for the target learner level.

##### 2.4.2.3 Examination consistency across the study period (2019–2025):

To ensure valid comparisons of academic performance between the historical control group (2019–2022) and the experimental group (2023–2025), we maintained strict consistency in examination content, format, difficulty, and grading criteria across the entire study period. Specifically, the examination format and scope were identical for both groups, with all test items drawn from the same validated item bank. Importantly, examination papers of similar difficulty from this bank were used for both groups, thereby minimizing the impact of examination difficulty differences on the observed outcomes.

To assess whether examination difficulty remained stable over the study period, we used an independent group of test-takers. Specifically, we recruited 18 laboratory interns from the Department of Clinical Laboratory who were not involved in the study as our calibration cohort. Each of the examination papers was administered to this cohort.

#### 2.4.3 Student feedback

Student perceptions were collected regarding their satisfaction with the teaching method, its perceived impact on their professional confidence, and its effectiveness in bridging the gap between theoretical knowledge and clinical practice. The structured questionnaires used to assess satisfaction (via an end-of-curriculum evaluation) are provided as Supplementary Material S4 Part A. The satisfaction survey consisted of 8 items covering overall satisfaction, perceived learning effectiveness, development of clinical reasoning skills, and professional identity formation. Prior to formal administration, questionnaires were pilot-tested for clarity and face validity with a small cohort of students (*n* = 5).

To assess the quality and consistency of facilitation across CBL sessions, students completed a Facilitator Evaluation Form at the end of each session. The form consisted of 5 items rated on a 5-point Likert scale (1 = strongly disagree to 5 = strongly agree), measuring: (1) clarity of learning objectives, (2) encouragement of active participation, (3) promotion of clinical reasoning through questioning, (4) provision of useful feedback without direct answers, and (5) overall satisfaction with facilitator guidance. The complete form is provided in Supplementary Material S4 Part B.

For the historical control group (traditional lecture format), parallel versions of the satisfaction survey (Supplementary Material S4 Part A) and instructor evaluation form (Supplementary Material S4 Part B) were used. The parallel instruments maintained identical dimensions and response scales to enable direct comparison between the two groups.

### 2.5 Statistical analysis

Data were analyzed using SPSS 22.0. Continuous variables (age, prior academic performance, examination scores, and engagement scores) were compared between groups using independent samples t-tests and presented as mean ± SD. Categorical variables (gender) were analyzed using chi-square tests. Given that the historical control period (2019–2022) coincided with the COVID-19 pandemic, which may have disrupted medical education, an analysis of covariance (ANCOVA) was performed as a sensitivity analysis to adjust for potential pandemic-related confounding (training time lost, proportion of online teaching). Linear regression was used to assess the trend in examination difficulty over time, with academic year as the independent variable and mean item difficulty as the dependent variable. A *P* < 0.05 was noted for descriptive purposes, recognizing that statistical significance thresholds are not strictly applicable given the quasi-experimental, exploratory design.

## 3 Results

### 3.1 Construction of the clinical laboratory diagnostics case library

A standardized teaching case library was successfully constructed following the principles of systematization, normalization, practicality, and timeliness. Cases were sourced from real clinical data through the Laboratory Information System (LIS) and Hospital Information System (HIS), and underwent double review by laboratory medicine and clinical experts. As shown in Table 6, the case library comprises 28 standardized teaching cases organized into six major subspecialty modules. The distribution of cases was demonstrated that Clinical Hematology and Clinical Biochemistry had the highest number of cases (6, 21.4%), followed by Clinical Immunology and Clinical Body Fluids Analysis (5, 17.9%), Clinical Microbiology and Clinical Molecular Biology (3, 10.7%). This distribution reflects the relative instructional emphasis of each subspecialty in the overall curriculum. All 28 cases were utilized during the CBL curriculum. This structure ensures comprehensive coverage of the core curriculum. The cases are continuously updated to reflect the latest diagnostic criteria and technological advancements, with a dynamic update mechanism that incorporates new evidence and retires outdated content.

**TABLE 6:** Organizational framework of the teaching case library for clinical laboratory diagnostics.

Serial number	Subspecialty	Case type	Core knowledge points involved in clinical laboratory testing
1	Clinical hematology	Infection	White blood cell counts and differential
		Anemia	Red blood cell counts and hemoglobin concentration
		Thrombocytopenia	Platelet count and morphological examination
		Various types of leukemia	Blood cell morphological examination
		Coagulation abnormalities	Hemostasis and thrombosis-related tests
		Malaria	Microscopic examination for malaria parasites
2	Clinical body fluid analysis	Urinary tract infection	Physical and chemical examination of urine and sediment microscopy
		Enteritis	Routine stool examination
		Parasitic infection	Microscopic examination for parasites
		Meningitis	Routine biochemical examination of cerebrospinal fluid
		Pleural effusion	Routine biochemical examination of serous cavity fluid
3	Clinical biochemistry	Diabetes mellitus	Glucose metabolism tests
		Hyperlipidemia	Lipid metabolism tests
		Acute myocardial infarction	Myocardial injury tests
		Chronic renal insufficiency	Renal function tests
		Liver cirrhosis	Liver function tests
		Multiple myeloma	Serum protein electrophoresis: M protein
4	Clinical immunology	Autoimmune disease	Autoantibody testing
		Active tuberculosis	Mycobacterium tuberculosis immunological testing: T-SPOT.TB test
		Syphilis	Infection immunology tests
		Tumor	Tumor marker tests
		Hyperthyroidism	Hormone tests: Thyroid hormones, thyroid-stimulating hormone, etc.
5	Clinical microbiology	Bacterial infection	Microbial staining, bacterial isolation, culture, identification, and antimicrobial susceptibility testing
		Fungal infection	Microbial staining, fungal isolation, culture, and identification
		Bloodstream infection	Blood culture and identification
6	Clinical molecular biology	Respiratory tract infection	Respiratory pathogen nucleic acid testing
		Chronic viral hepatitis	Hepatitis virus nucleic acid testing
		Lung cancer	EGFR gene mutation analysis

### 3.2 Participant baseline characteristics

The baseline characteristics of the 36 participants are summarized in Table 7. The historical control group and experimental group were comparable in terms of age at enrollment (24.9 ± 4.0 vs. 24.5 ± 2.7 years, *P* = 0.73), gender distribution (83.3% vs. 77.8% female, *P* = 0.67), and prior academic performance (entrance exam scores: 81.6 ± 9.9 vs. 83.3 ± 10.2, *P* = 0.66). No significant differences were observed between the two groups for any baseline variable, supporting the validity of the historical control design.

**TABLE 7:** Participants baseline characteristics.

Parameter	Historical control group (*n* = 18)	Experimental group (*n* = 18)	*P*-value
Enrollment period	2019–2022	2023–2025	
Teaching method	Traditional lecture-based teaching (TLT)	Case-based learning (CBL) with case library	
Age at enrollment (years, mean ± SD)	24.9 ± 4.0	24.5 ± 2.7	0.73
Gender (female, n, %)	15 (83.3%)	14 (77.8%)	0.67
Prior academic performance (mean ± SD)	81.6 ± 9.9	83.3 ± 10.2	0.66

### 3.3 Implementation of case-based learning: a systemic lupus erythematosus (SLE) case demonstration

To illustrate the practical application of the case library in teaching, we present the detailed implementation process using a typical autoimmune disease case—systemic lupus erythematosus (SLE)—from the Clinical Immunology module.

#### 3.3.1 Teaching objectives

This case, derived from a real clinical scenario, was designed around the diagnostic workup of SLE. The specific teaching objectives were: (1) to reinforce foundational knowledge by deepening understanding of autoantibody testing and clarifying the clinical significance of relevant laboratory tests; (2) to enhance core skills in result interpretation and abnormal report analysis; (3) to cultivate clinical reasoning by guiding students to establish “support/exclude” differential diagnostic logic based on laboratory evidence; (4) to strengthen practical application by helping students understand the role of laboratory results in treatment planning and disease monitoring; and (5) to expand professional competence by integrating recent literature and clinical guidelines.

#### 3.3.2 Teaching procedure

The CBL implementation followed a structured three-stage process: pre-class preparation, in-class execution (five stages), and post-class evaluation (Figure 1).

##### 3.3.2.1 Pre-class preparation

One week before the session, students were provided with the SLE case materials, relevant literature on SLE pathogenesis and laboratory diagnosis, and guiding questions: “What is the pathogenesis of SLE?” “What is the laboratory diagnostic criteria for SLE?” “Which laboratory tests are useful for monitoring SLE disease activity?”

##### 3.3.2.2 In-class implementation

Stage 1: Foundational Knowledge Reinforcement and Test Selection. The session began with a clinical scenario introduction—e.g., “Sunlight Becomes “Forbidden”: The Interdisciplinary Diagnostic Journey of a 26-Year-Old Female Patient”—to establish the connection between clinical presentations and laboratory needs. Through textual descriptions and images of the patient’s symptoms, history, and physical findings, students were asked: “What are the possible etiologies for this patient presenting with fever, facial erythema, and arthralgia? Which laboratory tests should be prioritized?” This exercise enabled students to distinguish between screening tests and diagnostic tests, and to understand the rationale for test selection based on specific case characteristics.

Stage 2: Result Interpretation and Critical Analysis. Students analyzed laboratory data step by step, identifying abnormalities and their degree. They integrated multiple test results—such as antinuclear antibody (ANA), anti-dsDNA antibody titers, complement C3/C4 levels, complete blood count, erythrocyte sedimentation rate, and urinalysis—to explore their clinical correlations. Students were also guided to consider pre-analytical variables (e.g., sample quality) and analytical limitations, understanding that no single test is perfect and that comprehensive interpretation requires integration of multiple data sources.

Stage 3: Clinical Logic Construction—From Laboratory Data to Diagnosis. Students formulated diagnostic hypotheses based on abnormal findings (e.g., “Fever + facial erythema + ANA positivity suggests possible autoimmune disease”). They then gathered evidence supporting or refuting each hypothesis, such as high-titer anti-dsDNA antibodies and anti-Sm antibody positivity supporting SLE diagnosis. By excluding other autoimmune diseases (e.g., rheumatoid arthritis), students derived the most likely diagnosis. This stage also included simulated communication with clinicians to practice reporting and consultation skills.

Stage 4: Value of Laboratory Testing in Disease Management and Monitoring. Students analyzed how laboratory results influence treatment decisions and identified dynamic monitoring indicators (e.g., complement C3/C4, anti-dsDNA antibodies, ESR, CRP) for assessing disease activity and guiding treatment adjustments. The “treatment-testing-adjustment” logic was emphasized.

Stage 5: Competency Enhancement and Knowledge Extension. Potential biases in the diagnostic process should be considered, such as over-reliance on a single test (8). By comparing autoimmune antibody profiles of SLE and rheumatoid arthritis, differential diagnostic thinking was reinforced. The discussion was extended to incorporate recent advances and clinical guidelines, including the “Chinese guideline for the diagnosis and treatment of systemic lupus erythematosus (2025 edition) (9)” and expert consensus on antinuclear antibody testing (10), promoting integration of clinical reasoning with cutting-edge knowledge.

##### 3.3.2.3 Post-class evaluation

Student learning outcomes, engagement, and satisfaction were assessed using questionnaires and a standardized teaching evaluation form. Data analysis focused on whether students achieved the teaching objectives and mastered relevant content. Participation and satisfaction were tracked to optimize learning motivation and foster professional commitment.

### 3.4 Educational outcomes of CBL implementation

Observed outcomes associated with the CBL model fell into three main categories: engagement, academic performance, and student feedback. Given the quasi-experimental design with historical controls, inferences about statistical significance should be made with caution. These findings are exploratory.

#### 3.4.1 Enhanced student engagement and participation:

The implementation of the CBL model led to a marked increase in classroom activity. Student engagement was evaluated using instructor ratings and student self-reported surveys. Instructor ratings on a 5-point Likert scale (1 = very low to 5 = very high) showed that 66.6% of students in the CBL group achieved a “high engagement” level (score ≥ 4), compared to only 33.3% of students in the control group. Student self-reported engagement, measured using a post-session survey (5-point Likert scale, 1 = strongly disagree to 5 = strongly agree), also revealed higher scores in the CBL group. The mean composite engagement score (mean of five Likert items) was 4.5 ± 0.4 in the CBL group versus 3.2 ± 0.6 in the control group (*P* < 0.01), representing a 1.3-point increase. These quantitative findings indicate a substantial improvement in active participation, consistent with instructors’ observation that the interactive, small-group format encouraged students to “dare to speak and dare to make decisions.”

#### 3.4.2 Improved academic performance

Before comparing academic performance between the two groups, we first confirmed that examination difficulty remained relatively stable across the study period, as supported by linear regression analysis (slope = 0.0048, *R*^2^ = 0.20, *P* = 0.31; see Supplementary Material S5).

**FIGURE 2:**
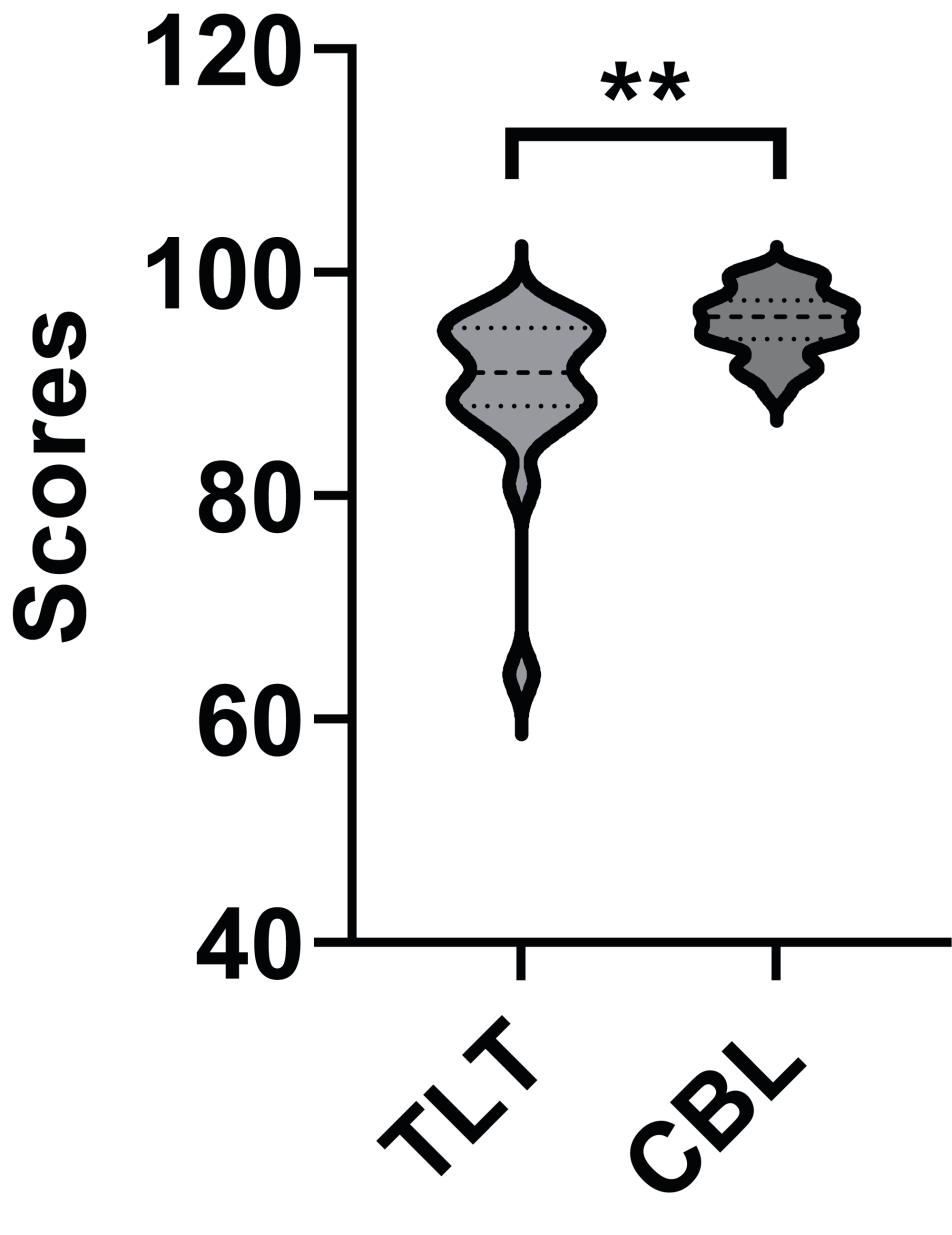
Comparison of end-of-rotation examination scores between the case-based learning (CBL) and traditional lecture-based teaching (TLT) groups. Students in the CBL group achieved higher examination scores (95.4 ± 2.9) than those in the TLT group (89.6 ± 7.5), as assessed by an independent-samples *t*-test (*t* = 3.26, *P* = 0.004). Data are presented as mean ± standard deviation. **P < 0.01.

Analysis of end-of-rotation examination scores revealed that the CBL group performed better than the traditional lecture-based teaching (TLT) group. As shown in Figure 2, students in the CBL group scored an average of 95.4 ± 2.9 points, which was 5.8 points higher than the TLT group’s average of 89.6 ± 7.5 points (*P* = 0.004). This difference suggests that CBL may be more effective in facilitating the internalization of theoretical knowledge and the application of clinical reasoning, although causal inferences cannot be drawn from this quasi-experimental design. Notably, the low standard deviation in the CBL group suggests a clustering of scores near the upper range, potentially reflecting a ceiling effect of the current assessment. Future studies should consider including more challenging, application-level items to better differentiate performance among high-achieving students.

##### 3.4.2.1 Sensitivity analysis: accounting for COVID-19 pandemic confounding

The historical control period (2019–2022) coincided with the COVID-19 pandemic, which caused major disruptions in hospital access, clinical laboratory routines, and medical education worldwide. To assess whether the observed difference in academic performance could be attributed to pandemic-related disruptions rather than the CBL intervention, we conducted a sensitivity analysis.

Pandemic-related variables were extracted from institutional records for each academic year. As expected, the historical control group experienced more pandemic-related disruption than the experimental group, including greater loss of training time due to access restrictions and a higher proportion of online teaching (*P* < 0.001 for both comparisons). Sensitivity analysis using ANCOVA, adjusting for these pandemic-related variables (e.g., training time lost, proportion of online teaching), supported our primary conclusion that the CBL intervention, rather than pandemic-related factors, was responsible for the observed improvement in student performance (*P* < 0.01).

##### 3.4.2.2 Positive student feedback and professional development

###### 3.4.2.2.1 Quantitative findings

Student evaluations of the CBL teaching method and facilitator guidance were positive overall. The mean ratings for all eight teaching method items were consistently high on the 5-point Likert scale, with the highest-rated items being those related to improved ability to interpret laboratory data and enhanced clinical reasoning skills. For facilitator guidance, mean ratings were also uniformly high, with the encouragement of active participation receiving the highest score. All items scored above the scale midpoint, suggesting generally favorable student perceptions.

###### 3.4.2.2.2 Qualitative themes

Analysis of open-ended responses revealed three overarching themes.

Reduced cognitive load: Students consistently reported that working with real cases alleviated the anxiety associated with applying theoretical knowledge to clinical practice. One student commented, “CBL made the transition from textbooks to clinical practice much less intimidating.”

Increased professional confidence: Students noted that collaborative problem-solving and facilitator-guided discussions helped them build confidence in making diagnostic decisions. A representative comment: “I feel more prepared to interpret actual patient results now.”

Strengthened professional identity: The immersive case approach fostered a sense of belonging to the laboratory medicine profession. As one student wrote, “For the first time, I could really see myself as a future laboratory physician.”

Overall, the majority of students expressed overall satisfaction with the CBL method and indicated they would recommend it for future professional master’s students. Facilitator guidance received comparable endorsement, suggesting that effective facilitation was integral to the success of the CBL curriculum. Thus, the structured, case-based approach demonstrated strong potential in cultivating clinical thinking, enhancing problem-solving skills, and supporting the psychological development of professional master’s students in Clinical Laboratory Diagnostics.

## 4 Discussion

The construction and implementation of a standardized teaching case library for Clinical Laboratory Diagnostics represents a potentially valuable advancement in postgraduate medical education, particularly for professional master’s degree students training to become laboratory physicians. This study demonstrates that a systematically developed, CBL-integrated case library may help bridge the critical gap between theoretical knowledge and clinical practice, offering a potential approach to addressing several longstanding challenges in laboratory medicine education.

The primary goal of professional degree postgraduate education in clinical laboratory diagnostics is to cultivate laboratory physicians with robust clinical practice competence and innovative capacity. Our findings confirm that a well-constructed case library, organized across six core subspecialty modules (hematology, body fluids, biochemistry, immunology, microbiology, and molecular biology), provides a comprehensive framework for achieving this objective (11). The structured approach ensures that students encounter a balanced representation of common diseases, typical cases, and complex diagnostic scenarios within a limited training period, thereby accelerating the accumulation of clinical experience. This aligns with the broader educational principle that systematic exposure to real-world cases facilitates the transformation of perceptual knowledge into structured cognitive frameworks.

The implementation of CBL using our case library demonstrated marked improvements in students’ clinical reasoning abilities (12). Our structured, five-stage CBL framework explicitly targets the incremental development of specific clinical competencies essential for laboratory physicians. During Stage 1, by correlating clinical presentations with laboratory test choice, students were trained to formulate test selection rationale—a skill that requires distinguishing screening from diagnostic tests based on clinical scenarios rather than rote memorization. In Stage 2, systematic analysis of laboratory data—integrating autoantibody profiles, complement levels, and hematological parameters—cultivated students’ ability to perform interpretation of conflicting or complex results, recognizing that no single test is infallible and that pattern recognition across multiple data points is critical. Stage 3 advanced this process by requiring students to construct evidence-based diagnostic hypotheses, explicitly weighing supportive versus contradictory laboratory evidence, thereby simulating the differential diagnosis process in real-world practice. Stage 4 extended clinical reasoning beyond diagnosis into therapeutic monitoring and disease management, where students learned to identify dynamic indicators (e.g., anti-dsDNA antibodies, complement C3/C4) and understand their roles in guiding treatment adjustments. Finally, Stage 5 fostered critical reflection on diagnostic biases and integration of contemporary guidelines and literature, preparing students to practice evidence-based laboratory medicine practice. The staged teaching approach—progressing from foundational knowledge reinforcement and test selection, through result interpretation and critical analysis, to clinical logic construction and therapeutic monitoring—mirrors the natural cognitive progression of clinical decision-making (13). By deliberately scaffolding these competencies across the five stages, our CBL framework ensures that students not only acquire knowledge but also develop the nuanced clinical judgment required for independent practice.

The observed improvements in student performance may be understood through relevant educational theories, though direct measurement of psychological constructs was not undertaken. The structured, five-stage format may effectively reduce the cognitive load associated with the theory-to-practice transition—an interpretation consistent with Cognitive Load Theory, which posits that well-designed learning materials reduce extraneous cognitive load. However, as no specific scales were used to measure cognitive load directly, this remains a theoretical explanation rather than an empirically established outcome.

Similarly, the interactive, small-group format fostered a supportive learning environment that, based on student feedback, gradually built students’ professional confidence and willingness to collaborate (14). These findings align with the principles of Self-Determination Theory, particularly the satisfaction of needs for autonomy (freedom to explore case discussions), competence (mastery of interpretation skills), and relatedness (collaborative learning with peers). Again, because validated instruments were not administered to measure these psychological constructs, these links should be viewed as potential theoretical explanations rather than directly measured outcomes of the study.

Student reflections—such as “daring to speak and dare to make decisions”—indicate a progressive shift from passive acceptance to active decision-making, accompanied by strengthened professional identity (15, 16). These affective outcomes are particularly relevant for laboratory medicine professionals, who must confidently navigate the interface between laboratory technology and patient-facing care while maintaining effective interdisciplinary relationships.

The observed improvements in student engagement (a 1.3-point difference on a 5-point scale) and examination scores (a 5.8-point difference) are consistent with the possibility that this approach may enhance learning outcomes, aligning with previous case-based studies (12). However, given the quasi-experimental design with historical controls, these differences should be interpreted as descriptive indicators of effect magnitude rather than as confirmatory evidence of efficacy. Notably, the integration of simulated clinician communication into case discussions prepares students for their future roles on multidisciplinary healthcare teams, where the effective interpretation and communication of laboratory data are paramount.

Nevertheless, a ceiling effect in the CBL group’s examination scores warrants caution. As reported, the CBL group achieved a mean score of 95.4 (SD = 2.9) on a 100-point scale, indicating insufficient discriminative range to capture meaningful performance variation among high-achieving students. Consequently, the observed 5.8-point difference should be interpreted with caution, as this ceiling effect materially limits the interpretive weight of the examination outcome. The true pedagogical advantage of CBL may be either underestimated or misrepresented by an instrument that cannot adequately capture variation in higher-order learning. Therefore, the examination result should be viewed as a preliminary, descriptive indicator rather than a robust confirmatory measure of efficacy. Future studies should employ more challenging, application-level assessments (e.g., complex case analyses with open-ended components) to better differentiate performance among top-achieving learners. Until such refinements are made, the between-group difference should not be over-interpreted as a precise estimate of educational effect.

The closed-loop construction model—initial case library development, CBL implementation and evaluation, and subsequent case updating—ensures that the curriculum remains current with evolving medical knowledge and technology. The incorporation of recent clinical guidelines (e.g., the 2025 Chinese guideline for the diagnosis and treatment of systemic lupus erythematosus) and expert consensus statements into case design reflects a commitment to evidence-based, up-to-date education. This dynamic updating mechanism is essential in a field characterized by rapid technological innovation and evolving diagnostic criteria (17).

The successful application of this case library in clinical laboratory diagnostics has broader implications for the training of laboratory medicine students and multidisciplinary healthcare education. As laboratory medicine increasingly integrates with clinical decision-making, precision medicine, and digital health platforms, educational models must evolve accordingly. The competency-based framework developed in this study—emphasizing test selection rationale, result interpretation, critical analysis of interfering factors, and clinical correlation—provides a transferable template for other laboratory medicine subspecialties and related disciplines, particularly for case-based teaching of laboratory medicine students (18). Although this study was conducted at a single tertiary hospital, the case library was designed for broad applicability. Cases are mostly based on common diseases and routine laboratory findings. The library can be implemented with minimal technological requirements (paper-based), facilitating its adoption across different levels of laboratory medicine educational programs. For institutions with diverse student backgrounds, the progressive difficulty sequencing (easy, moderate, difficult) accommodates learners with varying levels of prior knowledge. However, institutions with substantially different learner populations (e.g., undergraduates, international students) may need to adapt case difficulty or supplement with foundational materials. Future multi-center studies should specifically examine the transferability of this case library across different institutional settings, learner populations, and resource contexts.

## 5 Limitations and future directions

Several limitations of this study should be acknowledged. First, the relatively small sample size and single-center design may limit generalizability, and the findings should therefore be considered preliminary. Second, the comparison with traditional teaching methods relied on historical controls rather than a randomized controlled design, introducing risks of temporal confounding. The control period (2019–2022) coincided with the COVID-19 pandemic, which caused global disruptions to medical education. Although sensitivity analyses adjusting for pandemic-related variables (e.g., training time lost, online teaching proportion) suggested that the observed CBL effect remained relatively stable, we cannot completely exclude residual confounding from unmeasured factors such as student psychological stress and faculty burnout. Additionally, historical controls may differ from the intervention group on unmeasured variables (e.g., motivation, study habits) that could influence outcomes. While baseline characteristics were comparable between groups (all *P* > 0.05), selection bias cannot be entirely ruled out. Third, a ceiling effect was observed in the CBL group’s examination scores (see Discussion for a detailed analysis). This limits the interpretive weight of the examination score outcome, and the observed between-group difference should therefore be interpreted with caution. Fourth, potential assessment bias should be considered, as the instructors who delivered the CBL intervention were also involved in evaluating student performance. To mitigate this risk, we implemented several measures: (1) standardized rubrics with answer keys; (2) anonymized examination responses; (3) blinded grading where feasible; and (4) anonymous online engagement surveys, inaccessible to instructors until after final grades were submitted. Nevertheless, complete elimination of assessment bias remains challenging in educational research. Fifth, long-term follow-up data on graduates’ clinical performance and career outcomes are not yet available, though initial employer feedback has been favorable.

Future research should focus on multi-center validation studies, assessment of long-term educational outcomes, and exploration of digital enhancements such as AI-assisted case generation and virtual patient simulations (19). Additionally, the integration of this case library with emerging digital health platforms and competency-based medical education frameworks warrants further investigation.

## 6 Conclusion

In conclusion, this single-center, quasi-experimental study suggests that a systematically constructed, standardized CBL-integrated case library for clinical laboratory diagnostics may be associated with improved postgraduate education outcomes, including enhanced clinical reasoning and problem-solving abilities. However, given the non-randomized design and small sample size, these hypothesis-generating findings should be interpreted as exploratory rather than confirmatory, and require independent validation. While promising, the generalizability of this study is constrained by its single-center design and limited sample size. Therefore, future multi-center studies with larger cohorts are essential to validate this model as a practical, scalable framework—one aligned with modern, multidisciplinary healthcare delivery and capable of serving as a transferable template for educational transformation across health professions.

## Data Availability

The raw data supporting the conclusions of this article will be made available by the authors, without undue reservation.
