# Supplementary material for: Bridging the theory-practice gap in laboratory medicine: development and application of a standardized case library for fostering clinical reasoning in postgraduate training
Source: Front Med (Lausanne). 2026 Jun 24;13:1834799. doi: 10.3389/fmed.2026.1834799 (PMC13342040; doi:10.3389/fmed.2026.1834799)
Supplement: Supplementary file 1 [file Supplementary_file_1.pdf]

**Supplementary Material S1**

# Standard Case Template for Clinical Laboratory Diagnostics

**Case ID:** [Subspecialty Code - Number]
**Case Title:** [Concise and engaging title, may include subtitle]
**Subspecialty:** ☐ Clinical Hematology ☐ Clinical Body Fluids ☐ Clinical Biochemistry ☐ Clinical Immunology ☐ Clinical Microbiology ☐ Clinical Molecular Biology
**Case Type:** ☐ Common Disease ☐ Complex/Rare Disease ☐ Misdiagnosis Case ☐ Typical Case ☐ Critical/ Emergency Case
**Difficulty Level:** ☐ Easy (Difficulty Index 4-5) ☐ Moderate (Difficulty Index 6-7) ☐ Difficult (Difficulty Index 8-9)
**Estimated Teaching Duration:** [ ] minutes

## 1. Learning Objectives

Upon completion of this case, students should be able to:

| No. | Learning Objectives (Use Bloom's taxonomy verbs) |
|---|---|
| 1 | [Knowledge level, e.g., describe/list/identify] |
| 2 | [Application level, e.g., apply/calculate/interpret] |
| 3 | [Analysis level, e.g., analyze/differentiate/compare] |
| 4 | [Synthesis/Evaluation level, e.g., integrate/evaluate/judge] |
| 5 | [Professionalism/Attitude level, e.g., demonstrate/develop] |

## 2. Keywords

[3-6 keywords separated by semicolons]

## 3. Clinical Case Data

### 3.1 Chief Complaint

[Primary symptom(s) + duration]

### 3.2 History of Present Illness

[Detailed description of onset, evolution of symptoms, and diagnostic/treatment history]

### 3.3 Past Medical History

[Past illnesses, medication history, allergies, family history]

### 3.4 Physical Examination Findings

[Vital signs, specialty-specific findings, positive physical signs]

### 3.5 Laboratory Data

| Test | Result | Reference Range | Interpretation |
|---|---|---|---|
| [Test name] | [Result] | [Reference range] | [↑/↓/Normal] |
| [Test name] | [Result] | [Reference range] | [↑/↓/Normal] |
| ... | ... | ... | ... |

### 3.6 Ancillary Tests

[Imaging, pathology, other special tests]

### 3.7 Diagnostic Summary

[Concise summary integrating key clinical, laboratory, and ancillary findings that support the final diagnosis or differential diagnoses. May include diagnostic criteria, clinical reasoning pathway, and conclusive diagnosis.]

**Teaching Note** – Initially Hidden:

- This section is intended to be hidden from learners during the initial case presentation and early discussion. Do not include this subsection in student versions of the case materials.
- Reveal only after students have completed their own diagnostic reasoning (e.g., post-discussion, as a handout, or instructor-led summary).
- For a student-facing version of this template, delete Section 3.7 entirely.

## 4. Guided Discussion Questions

| No. | Guided Question | Corresponding Objective |
|---|---|---|
| Q1 | [Foundational question] | [Objective No.] |

| No. | Guided Question | Corresponding Objective |
|---|---|---|
| Q2 | [Analytical question] | [Objective No.] |
| Q3 | [Integrative question] | [Objective No.] |
| Q4 | [Advanced/Extended question] | [Objective No.] |

## 5. Teaching Implementation Flow

| Stage | Content | Teaching Activity | Duration |
|---|---|---|---|
| Stage 1: Test Selection | [Content] | [Activity] | [min] |
| Stage 2: Result Interpretation | [Content] | [Activity] | [min] |
| Stage 3: Clinical Reasoning & Differential Diagnosis | [Content] | [Activity] | [min] |
| Stage 4: Treatment & Monitoring | [Content] | [Activity] | [min] |
| Stage 5: Knowledge Extension & Reflection | [Content] | [Activity] | [min] |

### Teaching Tips and Considerations

- Pre-class preparation: [Suggested readings or materials for students]
- Classroom organization: [Suggested group size, role assignments]
- Common pitfalls: [Cognitive biases students may encounter]
- Interdisciplinary integration: [Other specialists who could be invited]

## 6. Key Takeaways

| No. | Key Takeaway |
|---|---|
| 1 | [Takeaway 1] |
| 2 | [Takeaway 2] |

| No. | Key Takeaway |
|---|---|
| 3 | [Takeaway 3] |
| ... | ... |

## 7. References


1. [Reference 1 (preferably guidelines/consensus statements)]
2. [Reference 2]
3. [Reference 3]


## 8. Validation Information

| Validation Dimension | Score/Conclusion | Validator |
|---|---|---|
| Content Validity (4-point scale) | [≥3.5 = Pass] | [Expert name/title] |
| Pedagogical Validity | [Pass/Pass with revision] | [Medical education expert] |
| Face Validity (pilot testing) | [Pass/Pass with revision] | [Student representative] |

## 9. Case Management and Version Control

| Item | Information |
|---|---|
| Author(s)/Affiliation | [Name] / [Affiliation] |
| Reviewer(s) | [Name] |
| Date of Inclusion | [YYYY-MM-DD] |
| Version Number | [VX. Y] |
| Update History | [Description of changes and date] |
| Next Review Date | [YYYY-MM-DD] |

## 10. Appendices

### 10.1 Subspecialty Codes Reference

| Code | Subspecialty |
|---|---|
| HEM | Clinical Hematology |
| BOD | Clinical Body Fluids |
| BIO | Clinical Biochemistry |
| IMM | Clinical Immunology |
| MIC | Clinical Microbiology |
| MOL | Clinical Molecular Biology |

### 10.2 Difficulty Index Calculation

The difficulty index is calculated using the following formula:

**Difficulty Index = Average (Clinical Complexity + Data Volume + Cognitive Demand + Prior Knowledge)**

| Parameter | Description | Score (1-3) |
|---|---|---|
| Clinical Complexity | Number of differential diagnoses (1=simple, 3=complex) | 1-3 |
| Data Volume | Number of laboratory parameters to interpret (1=few, 3=many) | 1-3 |
| Cognitive Demand | Level of integration required (1=low, 3=high) | 1-3 |
| Prior Knowledge Required | Based on curriculum mapping (1=basic, 3=advanced) | 1-3 |

**Mean Score Range:** 1.0-3.0

| Difficulty Tier | Mean Score Range |
|---|---|
| Easy | 1.0-1.5 |

| Difficulty Tier | Mean Score Range |
|---|---|
| Moderate | 1.6-2.2 |
| Difficult | 2.3-3.0 |

### 10.3 Template Usage Instructions

| Item | Guidance |
|---|---|
| Case ID Format | Subspecialty code (2 or 3 letters) + hyphen + two-digit number (e.g., IMM-01, HEM-05) |
| Learning Objectives | Use action verbs from Bloom's taxonomy; ensure alignment with guided questions |
| Laboratory Data | Include both normal and abnormal values; specify units and reference ranges |
| Guided Questions | Progress from basic recall to higher-order thinking (Bloom's taxonomy) |
| Validation Standard | Content validity mean score ≥3.5/4.0 required for inclusion |
| Version Update | Annual review recommended; update based on latest guidelines and evidence |

**Supplementary Material S2**

# Ten-item facilitator fidelity checklist for CBL implementation

| Item | Description | Yes | No |
|---|---|---|---|
| **1** | Asked open-ended questions (rather than yes/no questions) | ☐ | ☐ |
| **2** | Allowed student-led discussion | ☐ | ☐ |
| **3** | Provided corrective feedback without giving direct answers | ☐ | ☐ |
| **4** | Encouraged student-to-student responses and debate | ☐ | ☐ |
| **5** | Adhered to the planned time stages of the session | ☐ | ☐ |
| **6** | Guided students to address all intended learning objectives | ☐ | ☐ |
| **7** | Identified and corrected common student misconceptions | ☐ | ☐ |
| **8** | Avoided providing correct answers prematurely | ☐ | ☐ |
| **9** | Used guided questions from the case to drive discussion | ☐ | ☐ |
| **10** | Systematically summarized key knowledge points during the wrap-up phase | ☐ | ☐ |

**Total fidelity score (sum of "Yes" responses):** ____ / 10

**Supplementary Material S3**

# **Student Engagement Questionnaires** (CBL Version)

**Post-Session Engagement Survey (CBL Format):** completed after each CBL session

**Instructions:** Please rate your agreement with the following statements based on today's CBL session. (1=Strongly Disagree, 2=Disagree, 3=Neutral, 4=Agree, 5=Strongly Agree)

| **Item** | **Statement** | **1** | **2** | **3** | **4** | **5** |
|---|---|---|---|---|---|---|
| **1** | I felt actively involved in today's case discussion. | □ | □ | □ | □ | □ |
| **2** | I contributed meaningfully to the group discussion. | □ | □ | □ | □ | □ |
| **3** | The case-based format helped me stay focused throughout the session. | □ | □ | □ | □ | □ |
| **4** | I felt comfortable expressing my opinions and asking questions. | □ | □ | □ | □ | □ |
| **5** | The facilitator encouraged active participation from all students. | □ | □ | □ | □ | □ |

**Open-ended question:**

What was the most helpful aspect of today's CBL session?

# **Student Engagement Questionnaires** (Traditional Lecture Version)

**Post-Session Engagement Survey (Traditional Lecture Format):** completed after each traditional lecture session

**Instructions:** Please rate your agreement with the following statements based on today's traditional lecture session. (1=Strongly Disagree, 2=Disagree, 3=Neutral, 4=Agree, 5=Strongly Agree)

| Item | Statement | 1 | 2 | 3 | 4 | 5 |
|---|---|---|---|---|---|---|
| 1 | I felt actively engaged during today's lecture. | □ | □ | □ | □ | □ |
| 2 | When opportunities arose, I contributed meaningfully to classroom interactions (e.g., answering questions or participating in discussions). | □ | □ | □ | □ | □ |
| 3 | The lecture format helped me stay focused throughout the session. | □ | □ | □ | □ | □ |
| 4 | I felt comfortable expressing my opinions and asking questions to the instructor. | □ | □ | □ | □ | □ |
| 5 | The instructor encouraged active participation from all students. | □ | □ | □ | □ | □ |

**Open-ended question:**

What was the most helpful aspect of today's lecture session?

**Supplementary Material S4**

# **Student Satisfaction Questionnaires** (CBL Version)

**Part A End-of-Curriculum Satisfaction Survey (CBL Format):** completed after the entire CBL curriculum

**Instructions:** Please rate your agreement with the following statements based on your overall experience with the CBL curriculum. (1=Strongly Disagree, 2=Disagree, 3=Neutral, 4=Agree, 5=Strongly Agree)

| Item | Statement | 1 | 2 | 3 | 4 | 5 |
|---|---|---|---|---|---|---|
| **1** | Overall, I was satisfied with the CBL teaching method. | □ | □ | □ | □ | □ |
| **2** | CBL helped me better understand the clinical application of laboratory tests. | □ | □ | □ | □ | □ |
| **3** | CBL improved my ability to interpret laboratory data. | □ | □ | □ | □ | □ |
| **4** | CBL enhanced my clinical reasoning skills. | □ | □ | □ | □ | □ |
| **5** | CBL reduced the difficulty of transitioning from theory to practice. | □ | □ | □ | □ | □ |
| **6** | CBL increased my confidence in making diagnostic decisions. | □ | □ | □ | □ | □ |
| **7** | CBL strengthened my professional identity as a future laboratory physician. | □ | □ | □ | □ | □ |
| **8** | I would recommend CBL for future professional master's students. | □ | □ | □ | □ | □ |

**Open-ended questions:**

1. What did you find most valuable about the CBL curriculum?

2. What suggestions do you have for improving the CBL curriculum?

**Part B Facilitator Evaluation Form (CBL Format):** completed after each CBL session

**Instructions:** Please rate your agreement with the following statements about today's facilitator. (1=Strongly Disagree, 2=Disagree, 3=Neutral, 4=Agree, 5=Strongly Agree)

| Item | Statement | 1 | 2 | 3 | 4 | 5 |
|---|---|---|---|---|---|---|
| **1** | The facilitator clearly explained the learning objectives. | □ | □ | □ | □ | □ |
| **2** | The facilitator encouraged active participation from all students. | □ | □ | □ | □ | □ |
| **3** | The facilitator asked questions that promoted clinical reasoning. | □ | □ | □ | □ | □ |
| **4** | The facilitator provided useful feedback without giving direct answers. | □ | □ | □ | □ | □ |
| **5** | Overall, I was satisfied with the facilitator's guidance. | □ | □ | □ | □ | □ |

# **Student Satisfaction Questionnaires** (Traditional Lecture Version)

**Part A End-of-Curriculum Satisfaction Survey (Traditional Lecture Format):** completed after the entire traditional lecture curriculum

**Instructions:** Please rate your agreement with the following statements based on your overall experience with the CBL curriculum. (1=Strongly Disagree, 2=Disagree, 3=Neutral, 4=Agree, 5=Strongly Agree)

| Item | Statement | 1 | 2 | 3 | 4 | 5 |
|---|---|---|---|---|---|---|
| **1** | Overall, I was satisfied with the traditional lecture teaching method. | □ | □ | □ | □ | □ |
| **2** | The lecture format helped me better understand the clinical application of laboratory tests. | □ | □ | □ | □ | □ |
| **3** | The lecture format improved my ability to interpret laboratory data. | □ | □ | □ | □ | □ |
| **4** | The lecture format enhanced my clinical reasoning skills. | □ | □ | □ | □ | □ |
| **5** | The lecture format reduced the difficulty of transitioning from theory to practice. | □ | □ | □ | □ | □ |
| **6** | The lecture format increased my confidence in making diagnostic decisions. | □ | □ | □ | □ | □ |
| **7** | The lecture format strengthened my professional identity as a future laboratory physician. | □ | □ | □ | □ | □ |
| **8** | I would recommend the traditional lecture format for future professional master's students. | □ | □ | □ | □ | □ |

**Open-ended questions:**

1. What did you find most valuable about the traditional lecture curriculum?

---

2. What suggestions do you have for improving the traditional lecture curriculum?

---

**Part B Facilitator Evaluation Form (Traditional Lecture Format):** completed after each traditional lecture session

**Instructions:** Please rate your agreement with the following statements about today's facilitator. (1=Strongly Disagree, 2=Disagree, 3=Neutral, 4=Agree, 5=Strongly Agree)

| Item | Statement | 1 | 2 | 3 | 4 | 5 |
|---|---|---|---|---|---|---|
| **1** | The instructor clearly explained the learning objectives. | □ | □ | □ | □ | □ |
| **2** | The instructor encouraged active participation from all students. | □ | □ | □ | □ | □ |
| **3** | The instructor asked questions that promoted clinical reasoning. | □ | □ | □ | □ | □ |
| **4** | The instructor provided useful feedback without giving direct answers. | □ | □ | □ | □ | □ |
| **5** | Overall, I was satisfied with the instructor's guidance. | □ | □ | □ | □ | □ |

**Supplementary Material S5**

## Examination difficulty consistency across the study period (2019-2025):

Mean item difficulty (p) was estimated using an independent calibration cohort of 18 laboratory interns who were not part of the study groups. This cohort completed all examination forms (both original and parallel) under standardized conditions. The difficulty index (p) was defined as the mean score divided by the total score for each item (range: 0–1), with higher values indicating easier items. The reported values represent the mean of item-level difficulty indices across all items within each year's examination paper. This calibration approach ensures that difficulty estimates are independent of the performance of the study groups, thereby enabling valid cross-year comparisons. The mean difficulty (p) was 0.711 for the historical control period (2019–2022, original forms) and 0.718 for the CBL intervention period (2023–2025, parallel forms), representing a difference of 0.007 (Table S1). Linear regression analysis showed no statistically significant trend in examination difficulty over the study period (slope = 0.0048, $R^2$ = 0.20, P = 0.31). These findings support the comparability of academic performance between the two groups.

Table S1. Mean item difficulty of examination papers (2019–2025) based on independent calibration cohort (n=18 laboratory interns)

| Year | Test Form | Mean Item Difficulty (p) |
|---|---|---|
| 2019 | Original | 0.684 |
| 2020 | Original | 0.735 |
| 2021 | Original | 0.712 |
| 2022 | Original | 0.714 |
| 2023 | Parallel | 0.685 |
| 2024 | Parallel | 0.728 |
| 2025 | Parallel | 0.742 |
